# Nutritional-metabolic risk stratification for delirium in surgical critically ill patients: joint associations of the triglyceride–glucose index and stress hyperglycemia ratio

**DOI:** 10.3389/fnut.2026.1875967

**Published:** 2026-09-09

**Authors:** Fengwei Yao, Linquan Wu, Enliang Li

**Affiliations:** Department of General Surgery, The Second Affiliated Hospital, Jiangxi Medical College, Nanchang University, Nanchang, Jiangxi, China

**Keywords:** delirium, machine learning, nutritional-metabolic biomarkers, stress hyperglycemia ratio (SHR), surgical intensive care unit (SICU)

## Abstract

**Background:**

Delirium is a frequent acute neuropsychiatric complication in critically ill surgical patients and is associated with poor clinical outcomes. Nutritional-metabolic disturbances, including insulin resistance and stress-related glycemic dysregulation, may contribute to delirium susceptibility. This study investigated the independent and joint associations of the triglyceride–glucose index (TyG) and stress hyperglycemia ratio (SHR) with delirium in critically ill surgical patients.

**Methods:**

Adult patients admitted to surgical or trauma intensive care units were included. Delirium was identified using the Confusion Assessment Method for the Intensive Care Unit. Multivariable logistic regression, restricted cubic spline analysis, receiver operating characteristic curve analysis, subgroup analysis, Boruta feature selection, machine learning models, and SHapley Additive exPlanations were applied.

**Results:**

Among 1,370 eligible patients, 198 (14.45%) developed delirium. After full adjustment, the highest SHR tertile was associated with a higher odd of delirium compared with the lowest tertile [odds ratio (OR) 3.18, 95% confidence interval (CI) 1.87–5.42; *p* < 0.001]. The highest TyG tertile was also associated with increased delirium odds (OR 1.87, 95% CI 1.13–3.11; *p* = 0.015). When analyzed as continuous variables, both SHR (OR 1.72, 95% CI 1.20–2.45; *p* < 0.001) and TyG (OR 1.93, 95% CI 1.39–2.67; *p* < 0.001) remained independently associated with delirium. Patients with concomitantly high TyG and high SHR had the greatest delirium odds (OR 3.57, 95% CI 2.00–6.36; *p* < 0.001). TyG showed a linear association with delirium, whereas SHR showed a nonlinear relationship. The combined TyG–SHR indicator showed moderate discrimination [area under the curve (AUC) 0.716]. The GBM model achieved an AUC of 0.853 (95% CI: 0.804–0.902) on the test set.

**Conclusion:**

The combined assessment of TyG and SHR may provide a novel approach for integrating chronic metabolic vulnerability and acute stress hyperglycemia, thereby improving delirium risk stratification in critically ill surgical and trauma patients.

## 1 Introduction

Delirium is a common acute brain dysfunction syndrome in critically ill surgical patients, principally defined by fluctuating changes in attention, state of awareness, and cognitive function (1, 2). Previous studies have shown that delirium is not only closely associated with prolonged mechanical ventilation, longer intensive care unit stays, and increased hospitalization costs, but may also lead to long-term cognitive decline and adverse clinical outcomes (3). It has therefore become an important issue in critical care medicine and perioperative management. Although the number of studies investigating risk factors for delirium has increased in recent years, its pathogenesis remains complex, involving multiple mechanisms such as neuroinflammation, oxidative stress, microcirculatory dysfunction, metabolic disturbances, and neurotransmitter imbalance (4), and there are still substantial limitations in its early identification and risk stratification (5).

The triglyceride-glucose index (TyG) and its derived indices are convenient integrated markers used in clinical practice to assess metabolic stress and insulin resistance (6, 7). TyG reflects lipid and glucose metabolic status and is closely associated with insulin resistance, endothelial dysfunction, chronic low-grade inflammation, and oxidative stress, all of which are also considered to be involved in the development and progression of delirium. In recent years, the application of TyG in critically ill populations has gradually increased, and several studies have demonstrated a positive association between TyG and delirium in patients after gastric surgery, after cardiac surgery, and in patients with sepsis (8–10). Notably, recent studies have reported that a higher TyG-BMI is associated with a lower short-term mortality risk in patients with cardiovascular disease (11), a finding that may reflect the influence of nutritional status, physiological reserve, and the so-called “obesity paradox” in critically ill patients (12, 13). However, interpreting adverse outcomes in critically ill patients solely on the basis of TyG or its derived indices remains limited by the insufficient assessment of acute stress states.

Abnormal glucose metabolism is one of the major biological bases of adverse outcomes in critically ill patients. Under acute stress conditions, marked hyperglycemia may occur as a result of sympathetic nervous system activation, inflammatory cytokine release, and aggravated insulin resistance. Previous investigations have demonstrated that high blood glucose alone is related with many negative outcomes in critical illness; however, it is difficult to distinguish the different risks attributable to poor chronic glycemic control from those attributable to acute stress hyperglycemia. Therefore, the stress hyperglycemia ratio (SHR), as an index adjusted for an individual’s long-term glycemic background (14), has attracted increasing attention in recent years. SHR can more accurately reflect the abnormal elevation of blood glucose relative to baseline metabolic status under acute stress conditions, and has been shown to be associated with delirium risk in elderly critically ill patients, patients after heart valve surgery, and patients with stroke (15–17).

Importantly, TyG and SHR reflect metabolic status from two distinct dimensions: chronic insulin resistance/basal metabolic abnormalities and acute stress-related glucose dysregulation. Theoretically, these two indices may provide complementary value in the development of delirium: TyG places greater emphasis on underlying metabolic vulnerability, whereas SHR better captures glycemic imbalance in the setting of acute pathological stress. However, studies investigating the joint associations of TyG and SHR with delirium risk remain very limited, particularly in critically ill surgical patients, a population at especially high risk.

Unlike previous studies that focused on either chronic metabolic dysfunction or acute stress hyperglycemia alone, the present study aimed to investigate the independent and joint associations of TyG and SHR with delirium in critically ill surgical and trauma patients. By integrating a marker of chronic metabolic vulnerability (TyG) with a marker of acute stress-related metabolic dysregulation (SHR), we sought to determine whether their combined assessment could provide enhanced risk stratification beyond either biomarker alone.

## 2 Methods

### 2.1 Research participants

This analysis was based on the MIMIC-IV database (version 3.1) (18). A researcher who had completed the CITI certification program and acquired approved database access (certificate No. 65083321) extracted the study data.

The inclusion and exclusion criteria: (1) adult patients aged >18 years with a first admission to the surgical ICU or trauma ICU; (2) Patients with lacking essential laboratory data needed for the estimation of SHR or TyG; and (3) patients who developed delirium within 24 h after ICU admission. Patients who developed delirium within 24 h after ICU admission were excluded to ensure that TyG and SHR measurements preceded the occurrence of delirium and to minimize potential reverse causality.

**Figure 1:**
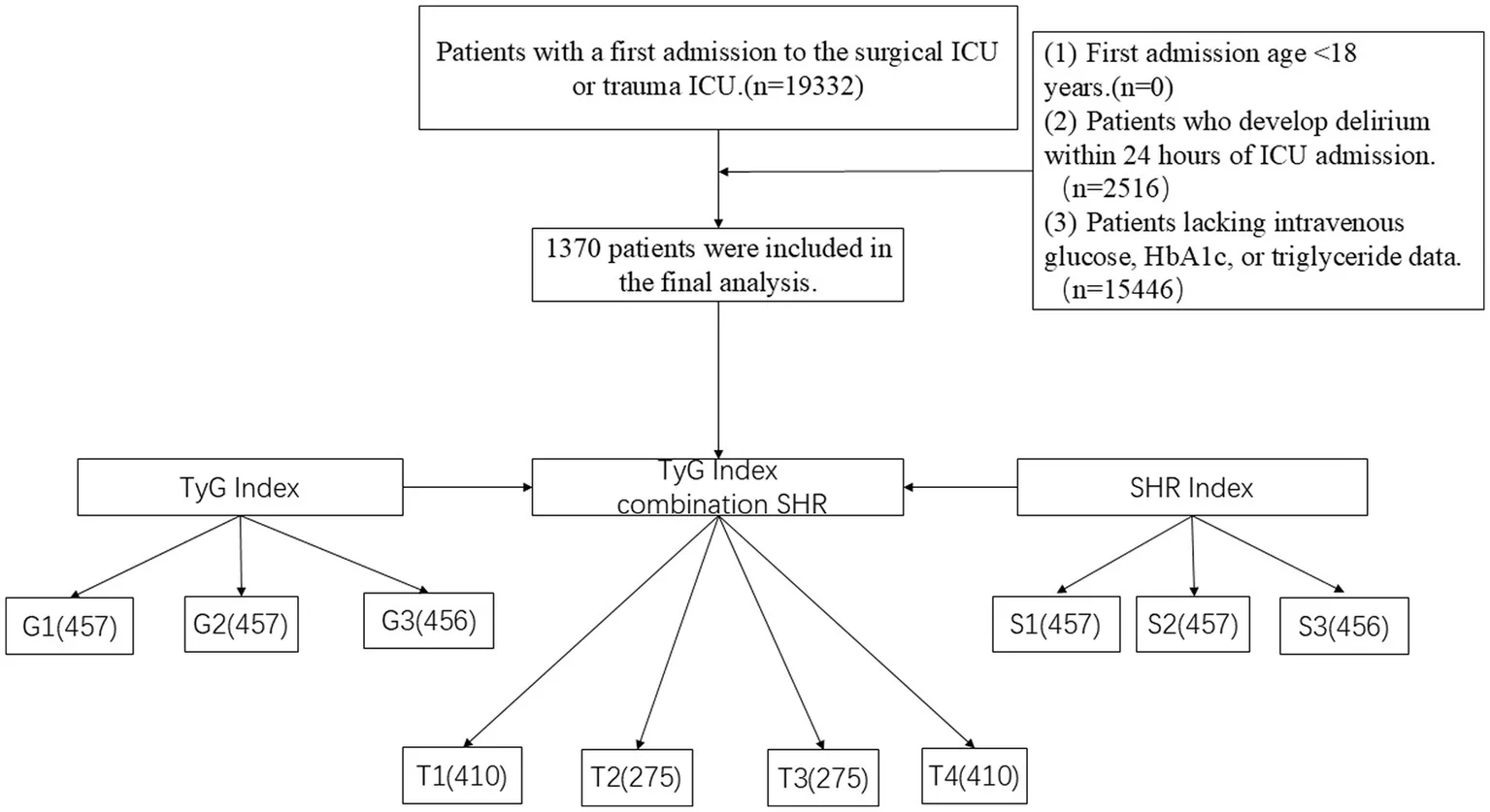
Flowchart of patient selection and grouping strategy based on TyG and SHR.

A total of 1,370 eligible patients were ultimately included. To preliminarily explore patient characteristics, the study population was first categorized according to the tertiles of TyG and SHR (Figure 1). Tertile-based groupings of TyG and SHR were used to evaluate their independent associations. Subsequently, TyG and SHR were dichotomized according to their median values, and these median-based binary categories were used to construct four joint exposure groups as follows:

T1 group: low TyG (≤8.846) + low SHR (≤1.088)
T2 group: high TyG (>8.846) + low SHR (≤1.088)
T3 group: low TyG (≤8.846) + high SHR (>1.088)
T4 group: high TyG (>8.846) + high SHR (>1.088)

### 2.2 Variable extraction and definitions

Data were extracted by running structured query language (SQL) scripts in Navicat Premium. Except for the critical exposure factors, all covariates were calculated based on the first recorded value within the first day of ICU admission. Delirium was assessed using the Confusion Assessment Method for the ICU (CAM-ICU), which evaluates four features: (1) acute change in mental status or a fluctuating course; (2) inattention; (3) disorganized thinking; and (4) altered level of consciousness. Delirium was considered positive when both features (1) and (2) were present, together with either feature (3) or (4) (19).

For the key exposure variables, specific measurement windows were defined according to their clinical significance and biological characteristics. The TyG index was calculated using the first glucose value and the first triglyceride value obtained during the index hospitalization. The TyG index was calculated using the following formula: TyG = ln [triglycerides (mg/dL) × glucose (mg/dL)/2] (10, 20). For SHR, considering that HbA1c reflects the average blood glucose level over the preceding 2–3 months and exhibits relatively little short-term fluctuation, the first HbA1c measurement obtained during the index hospitalization was used; the acute glycemic component was defined as the maximum glucose value within 24 h after ICU admission. The formula for SHR was as follows: SHR = maximum glucose value within 24 h after ICU admission/[(28.7 × HbA1c) − 46.7] (16, 21).

For variables with a missing proportion of less than 15%, multiple imputation was performed using the classification and regression tree (CART) method in the “mice” package in R (22).

Winsorization was applied to the first glucose value, maximum glucose value, triglycerides, and HbA1c using the “DescTools” package in R (23, 24). Winsorization was carried out using the corresponding percentile values for observations below the first percentile or above the 99th percentile. Visual examination using boxplots revealed that following this process, the impact of extreme numbers was much diminished (Supplementary Figure S1).

### 2.3 Statistical methods and analysis

Since logistic regression offers good interpretability and robustness in clinical data analysis and is appropriate for binary outcomes, it was chosen as the main statistical technique for this study.

To comprehensively identify potential confounding variables, we first used the Boruta algorithm for feature selection, and then included the identified variables in the multivariable models. In addition, for reasons of clinical relevance, several clinically important variables, such as age and sex, were retained *a priori* as adjustment covariates. Variance inflation factors were then calculated for all covariates to prevent multicollinearity. To assess the separate and combined relationships between SHR and TyG and delirium, four models were built:

- Model 1: only the exposure variables (SHR and TyG index);
- Model 2: additionally adjusted for sex, age, systolic blood pressure, weight, and oxygen saturation;
- Model 3: further adjusted for hemoglobin, platelet count, white blood cell count, red blood cell count, chloride, calcium, anion gap, bicarbonate, high-density lipoprotein, total cholesterol, low-density lipoprotein, Sequential Organ Failure Assessment score, and Simplified Acute Physiology Score II;
- Model 4: further adjusted for benzodiazepine use, dementia, opioid analgesic use, insulin use, cerebrovascular disease, vasoactive agent use, and continuous renal replacement therapy.

Restricted cubic spline (RCS) models with four knots were used to depict dose–response curves and to explore the dose–response relationships of TyG and SHR with delirium. Receiver operating characteristic (ROC) curves were generated to evaluate the discriminatory ability of TyG and SHR for delirium. Subgroup analyses were performed to assess the consistency of the associations of TyG and SHR across strata defined by sex, diabetes, heart failure, myocardial infarction, cerebrovascular disease, and age. To assess the robustness of our findings, two prespecified sensitivity analyses were performed. First, patients with severe anemia and those whose HbA1c, triglyceride, glucose, or maximum glucose measurements were obtained more than 24 h after ICU admission were excluded. TyG and SHR were subsequently recalculated using the remaining available measurements, and all primary analyses were repeated. Severe anemia was defined as a hemoglobin concentration below 60 g/L. Second, a complete-case analysis was conducted by excluding patients with missing values in any key study variable and repeating the primary analyses without multiple imputation. The same adjustment strategy as the primary analysis was applied in both sensitivity analyses.

Finally, machine learning-based predictive models were developed using a feature selection strategy aimed at optimizing predictive performance. These machine learning analyses were conducted as supplementary analyses to evaluate the incremental predictive contribution and relative importance of TyG and SHR rather than to establish a clinically applicable prediction model. To maintain consistency and interpretability across all algorithms, only the variables determined by the Boruta algorithm (explained above) were used as input features. 70% of the dataset was used to train each model, while the remaining 30% was used for evaluation. The training set was used for model development and hyperparameter tuning through repeated cross-validation, whereas all final model performance evaluations were conducted using the independent test set. The training set underwent five-fold cross-validation to avoid overfitting. The parameter settings of each model are shown in Supplementary Table S1. To improve the interpretability of the models and to identify the relative importance of each predictor for delirium risk, we introduced the SHapley Additive exPlanations (SHAP) method in the final model (25). Global feature importance plots were further generated to provide deeper insights into the model response patterns for SHR and TyG.

## 3 Results

### 3.1 Baseline clinical and demographic characteristics

A total of 1,370 patients were included in the analysis, of whom 198 (14.45%) developed delirium. Compared to patients who did not develop delirium, those who did had more severe acute metabolic disturbances, such as a higher heart rate, respiratory rate, creatinine, blood urea nitrogen, anion gap, triglycerides, glucose, maximum glucose, TyG index, and SHR. In addition, they had lower diastolic blood pressure, calcium, and bicarbonate. Moreover, multiple comorbidities and early ICU interventions also differed between the two groups, with higher rates of peripheral vascular disease, mild liver disease, diabetes, opioid analgesic use, insulin use, benzodiazepine use, vasoactive support, and continuous renal replacement therapy (CRRT) in the delirium group. In contrast, there were no significant differences between the groups in terms of age, gender, systolic blood pressure, oxygen saturation, hemoglobin, platelet count, sodium, HbA1c, or Glasgow Coma Scale score. Detailed baseline characteristics are presented in Table 1.

**Table 1:** Baseline clinical and demographic characteristics of patients with and without delirium.

Variables	Non-delirium (*n* = 1,172)	Delirium (*n* = 198)	*p*
Age (years)	68.31 ± 15.38	66.23 ± 14.61	0.076
SBP (mmHg)	141.44 ± 24.79	137.26 ± 29.06	0.058
DBP (mmHg)	76.32 ± 18.29	73.35 ± 19.59	0.037
Weight (Kg)	82.03 ± 38.65	87.21 ± 26.59	0.07
Temperature (°C)	36.75 ± 0.65	36.84 ± 0.87	0.096
Heart rate (times/min)	83.73 ± 18.60	89.57 ± 19.84	<0.001
Resp rate (times/min)	18.67 ± 5.40	20.51 ± 6.02	<0.001
SpO_2_ (%)	97.30 ± 2.71	97.02 ± 3.42	0.262
Hemoglobin (g/dL)	12.21 ± 2.12	11.88 ± 2.50	0.084
Platelet (×10^9^/L)	228.41 ± 93.76	214.40 ± 103.46	0.075
WBC (×10^9^/L)	10.53 ± 4.94	13.06 ± 6.09	<0.001
RBC (×10^12^/L)	4.07 ± 0.71	3.96 ± 0.86	0.095
Creatinine (mmol/L)	1.17 ± 1.09	1.56 ± 1.75	0.003
Chloride (mmol/L)	103.71 ± 5.26	102.86 ± 6.40	0.078
Calcium (mmol/L)	8.70 ± 0.74	8.48 ± 0.89	0.001
Potassium (mmol/L)	4.03 ± 0.64	4.17 ± 0.69	0.014
Sodium (mmol/L)	138.70 ± 4.28	138.30 ± 5.28	0.306
Anion gap (mmol/L)	14.40 ± 3.30	15.82 ± 4.57	<0.001
Bun (mmol/L)	20.65 ± 13.62	25.89 ± 23.66	0.003
Bicarbonate (mmol/L)	23.91 ± 3.68	22.06 ± 4.72	<0.001
PT (s)	14.19 ± 5.08	15.75 ± 8.30	0.011
APTT (s)	33.24 ± 18.03	36.11 ± 18.86	0.04
INR	1.28 ± 0.46	1.45 ± 0.76	0.003
HDL (mg/dL)	46.36 ± 17.00	41.31 ± 19.85	<0.001
TC (mg/dL)	162.60 ± 59.63	153.28 ± 48.81	0.037
LDL (mg/dL)	89.13 ± 41.25	81.10 ± 38.39	0.011
SOFA	1.02 ± 1.60	1.97 ± 2.75	<0.001
SAPSII	30.93 ± 11.41	39.09 ± 13.77	<0.001
HbA1c	6.25 ± 1.33	6.49 ± 1.76	0.07
TG (mg/dL)	137.66 ± 120.39	170.79 ± 156.07	0.005
Glucose (mg/dL)	136.82 ± 53.87	175.37 ± 82.34	<0.001
Glucose_Max (mg/dL)	149.63 ± 64.11	201.93 ± 97.48	<0.001
TyG	8.90 ± 0.69	9.27 ± 0.83	<0.001
SHR	1.15 ± 0.44	1.49 ± 0.64	<0.001
GCS	15.00 (14.00, 15.00)	15.00 (15.00, 15.00)	0.39
Myocardial infarct, *n* (%)			0.718
No	1,046 (89.25)	175 (88.38)	
Yes	126 (10.75)	23 (11.62)	
Congestive heart failure, *n* (%)			0.614
No	953 (81.31)	158 (79.80)	
Yes	219 (18.69)	40 (20.20)	
Peripheral vascular, *n* (%)			0.011
No	1,030 (87.88)	161 (81.31)	
Yes	142 (12.12)	37 (18.69)	
Cerebrovascular, *n* (%)			<0.001
No	258 (22.01)	73 (36.87)	
Yes	914 (77.99)	125 (63.13)	
Dementia, *n* (%)			0.45
No	1,137 (97.01)	194 (97.98)	
Yes	35 (2.99)	4 (2.02)	
Chronic pulmonary, *n* (%)			0.06
No	960 (81.91)	151 (76.26)	
Yes	212 (18.09)	47 (23.74)	
Rheumatic, *n* (%)			1
No	1,148 (97.95)	194 (97.98)	
Yes	24 (2.05)	4 (2.02)	
Peptic ulcer, *n* (%)			0.123
No	1,153 (98.38)	191 (96.46)	
Yes	19 (1.62)	7 (3.54)	
Mild liver, *n* (%)			<0.001
No	1,105 (94.28)	168 (84.85)	
Yes	67 (5.72)	30 (15.15)	
Diabetes all, *n* (%)			0.001
No	797 (68.00)	111 (56.06)	
Yes	375 (32.00)	87 (43.94)	
Paraplegia, *n* (%)			0.168
No	770 (65.70)	140 (70.71)	
Yes	402 (34.30)	58 (29.29)	
Renal disease, *n* (%)			0.963
No	990 (84.47)	167 (84.34)	
Yes	182 (15.53)	31 (15.66)	
Malignant cancer, *n* (%)			0.08
No	1,099 (93.77)	179 (90.40)	
Yes	73 (6.23)	19 (9.60)	
Gender, *n* (%)			0.943
F	642 (54.78)	109 (55.05)	
M	530 (45.22)	89 (44.95)	
Opioid analgesics used, *n* (%)			<0.001
No	838 (71.50)	70 (35.35)	
Yes	334 (28.50)	128 (64.65)	
Insulin used, *n* (%)			<0.001
No	355 (30.29)	18 (9.09)	
Yes	817 (69.71)	180 (90.91)	
Benzodiazepines used, *n* (%)			<0.001
No	902 (76.96)	108 (54.55)	
Yes	270 (23.04)	90 (45.45)	
Vasoactive used, *n* (%)			<0.001
No	1,016 (86.69)	122 (61.62)	
Yes	156 (13.31)	76 (38.38)	
CRRT, *n* (%)			<0.001
No	1,158 (98.81)	165 (83.33)	
Yes	14 (1.19)	33 (16.67)	

SpO₂, peripheral capillary oxygen saturation; SOFA, Sequential Organ Failure Assessment; SAPSII, Simplified Acute Physiology Score II; INR, international normalized ratio; PT, prothrombin time; APTT, activated partial thromboplastin time; BUN, blood urea nitrogen; RBC, red blood cell count; WBC, white blood cell count; SBP, systolic blood pressure; DBP, diastolic blood pressure; TG, triglyceride; GCS, Glasgow Coma Scale; TyG, triglyceride-glucose index; CRRT, continuous renal replacement therapy; HDL, high-density lipoprotein cholesterol; TC, total cholesterol; LDL, low-density lipoprotein cholesterol.

### 3.2 Boruta feature selection and multicollinearity control

**Figure 2:**
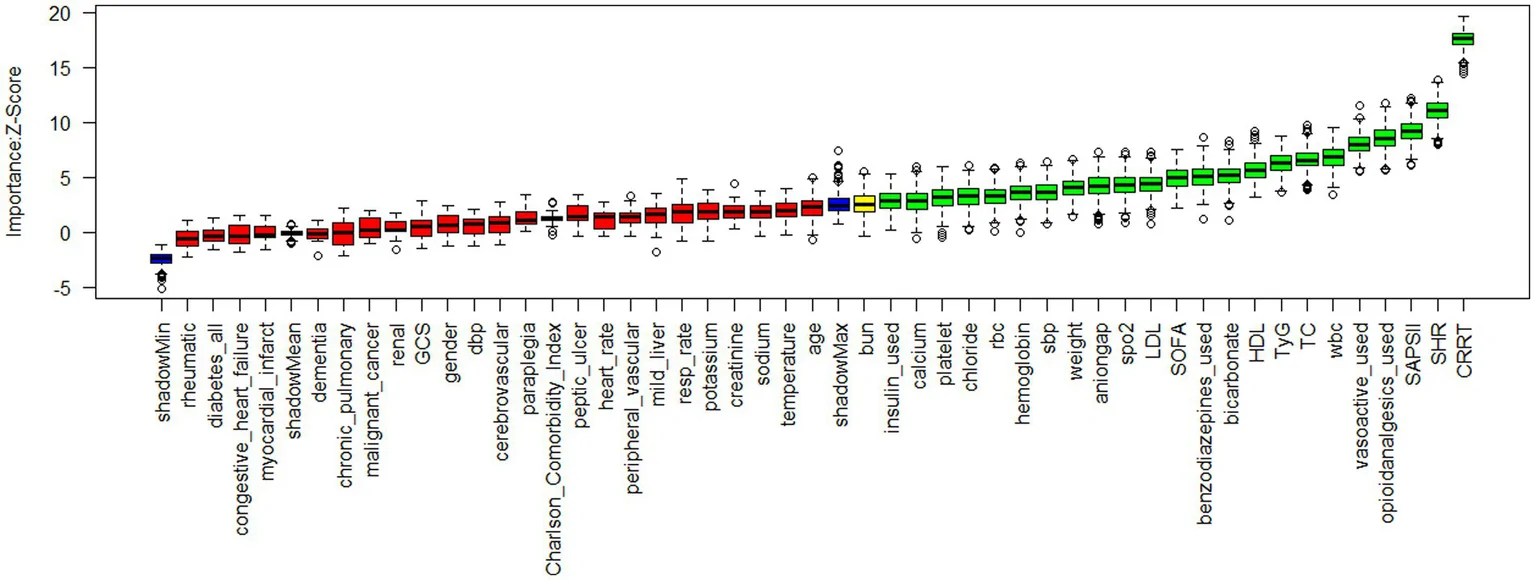
Boruta feature selection results and multicollinearity assessment.

To further identify the key features most closely associated with delirium, we performed feature selection using the Boruta algorithm based on all candidate variables. The variables entered into the Boruta analysis included comorbidities, demographic characteristics, vital signs, laboratory indicators, treatment-related factors, disease severity scores, and metabolic indices. After 500 iterations, the Boruta algorithm ultimately confirmed 23 important features, including SHR and TyG (Figure 2), while the remaining variables were classified as “Rejected” or “Tentative.” These results indicate that the confirmed features had relatively high and stable predictive value for discriminating between patients with and without delirium. Accordingly, the features selected by Boruta were incorporated into the subsequent machine learning model development.

On this basis, considering the clear clinical relevance of certain variables, we further included sex, age, cerebrovascular disease, and dementia in the multivariable logistic regression models to adjust for potential confounding. Variance inflation factor (VIF) analysis was then performed to assess multicollinearity among the variables entered into the models. Supplementary Table S2 summarizes the proportions of missing values before multiple imputation and the variance inflation factor (VIF) results for all key study variables. All VIF values were below 10, suggesting the absence of significant multicollinearity. Ultimately, all of the above variables were included in the logistic regression models for confounding adjustment.

### 3.3 Associations of the TyG index and SHR with delirium

In the independent association analysis of SHR (Table 2), using the lowest group (S1) as the reference, higher SHR levels were significantly associated with an increased odd of delirium, and this association remained stable across different adjustment models. In the fully adjusted model (Model 4), the risk of delirium increased by 2.16-fold (OR = 2.16, 95%CI: 1.26–3.72, *p* = 0.005) and 3.18-fold (OR = 3.18, 95%CI: 1.87–5.42, *p* < 0.001) in the S2 and S3 groups, respectively. When SHR was analyzed as a continuous variable, it also showed a significant positive association with delirium odd (OR = 1.72, 95%CI: 1.20–2.45, *p* < 0.001). In addition, trend analysis showed that the risk of delirium increased progressively with increasing SHR categories (*p* for trend <0.001). Overall, although the effect size was attenuated after multivariable adjustment, the positive association between SHR and delirium remained robust.

**Table 2:** Association between SHR tertiles and delirium risk in critically ill surgical patients.

Variables	Model 1		Model 2		Model 3		Model 4	
	OR (95%CI)	*p*	OR (95%CI)	*p*	OR (95%CI)	*p*	OR (95%CI)	*p*
S								
1	1.00 (Reference)		1.00 (Reference)		1.00 (Reference)		1.00 (Reference)	
2	2.80 (1.70 ~ 4.61)	<0.001	2.92 (1.76 ~ 4.85)	<0.001	2.44 (1.45 ~ 4.10)	<0.001	2.16 (1.26 ~ 3.72)	0.005
3	6.44 (4.03 ~ 10.29)	<0.001	6.55 (4.07 ~ 10.54)	<0.001	3.90 (2.35 ~ 6.47)	<0.001	3.18 (1.87 ~ 5.42)	<0.001
SHR	2.99 (2.28 ~ 3.93)	<0.001	2.99 (2.27 ~ 3.96)	<0.001	1.85 (1.34 ~ 2.56)	<0.001	1.72 (1.20 ~ 2.45)	<0.001
*p* for trend	13.05 (7.19 ~ 23.67)	<0.001	13.00 (7.13 ~ 23.69)	<0.001	5.89 (3.06 ~ 11.33)	<0.001	4.46 (2.22 ~ 8.98)	<0.001

OR, odds ratio; CI, confidence interval.

Model 1: Crude.

Model 2: Adjust: gender, age, sbp, weight, SpO₂.

Model 3: Adjust: gender, age, sbp, weight, SpO₂, hemoglobin, platelet, WBC, RBC, chloride, calcium, aniongap, bicarbonate, HDL, TC, LDL, SOFA, SAPSII.

Model 4: Adjust: cerebrovascular, dementia, gender, opioidanalgesics_used, insulin_used, benzodiazepines_used, vasoactive_used, CRRT, age, sbp, weight, SpO₂, hemoglobin, platelet, WBC, RBC, chloride, calcium, aniongap, bicarbonate, HDL, TC, LDL, SOFA, SAPSII.

A similar trend was also observed in the independent association analysis of the TyG index (Table 3). Using the lowest group (G1) as the reference, patients in the G3 group had a significantly increased odd of delirium in the fully adjusted model (OR = 1.87, 95%CI: 1.13–3.11, *p* = 0.015), whereas the association in the G2 group was no longer statistically significant after full adjustment (OR = 1.32, 95%CI: 0.82–2.12, *p* = 0.253). When TyG was analyzed as a continuous variable, it remained independently associated with delirium odd (OR = 1.93, 95%CI: 1.39–2.67, *p* < 0.001). Trend analysis further demonstrated that the odd of delirium increased significantly with increasing TyG levels (*p* for trend <0.001). These findings suggest that higher TyG levels were independently and positively associated with delirium occurrence.

**Table 3:** Association between TyG tertiles and delirium risk in critically ill surgical patients.

Variables	Model 1		Model 2		Model 3		Model 4	
	OR (95%CI)	*p*	OR (95%CI)	*p*	OR (95%CI)	*p*	OR (95%CI)	*p*
G								
1	1.00 (Reference)		1.00 (Reference)		1.00 (Reference)		1.00 (Reference)	
2	1.46 (0.96 ~ 2.23)	0.074	1.48 (0.97 ~ 2.25)	0.068	1.40 (0.89 ~ 2.19)	0.141	1.32 (0.82 ~ 2.12)	0.253
3	2.67 (1.81 ~ 3.94)	<0.001	2.59 (1.75 ~ 3.85)	<0.001	2.00 (1.25 ~ 3.21)	0.004	1.87 (1.13 ~ 3.11)	0.015
TyG	1.92 (1.58 ~ 2.33)	<0.001	1.89 (1.54 ~ 2.32)	<0.001	1.94 (1.43 ~ 2.64)	<0.001	1.93 (1.39 ~ 2.67)	<0.001
*p* for trend	2.11 (1.59 ~ 2.79)	<0.001	2.05 (1.54 ~ 2.73)	<0.001	1.67 (1.18 ~ 2.36)	<0.001	1.60 (1.10 ~ 2.32)	<0.001

OR, odds ratio; CI, confidence interval.

Model 1: Crude.

Model 2: Adjust: gender, age, sbp, weight, SpO₂.

Model 3: Adjust: gender, age, sbp, weight, SpO₂, hemoglobin, platelet, WBC, RBC, chloride, calcium, aniongap, bicarbonate, HDL, TC, LDL, SOFA, SAPSII.

Model 4: Adjust: cerebrovascular, dementia, gender, opioidanalgesics_used, insulin_used, benzodiazepines_used, vasoactive_used, CRRT, age, sbp, weight, SpO₂, hemoglobin, platelet, WBC, RBC, chloride, calcium, aniongap, bicarbonate, HDL, TC, LDL, SOFA, SAPSII.

Joint group analysis further showed that concurrent elevation of TyG and SHR was associated with an increased odd of delirium (Table 4). Using the joint lowest-risk group (T1) as the reference, the risk of delirium increased by 2.06-fold (OR = 2.06, 95%CI: 1.06–4.01, *p* = 0.033), 2.39-fold (OR = 2.39, 95%CI: 1.31–4.37, *p* = 0.005), and 3.57-fold (OR = 3.57, 95%CI: 2.00–6.36, *p* < 0.001) in the T2, T3, and T4 groups, respectively, in the fully adjusted model. As the joint exposure category increased, the OR values exhibited a stepwise upward trend, suggesting that concurrent abnormalities in TyG and SHR may exert a cumulative effect on delirium occurrence. To formally assess effect modification, a multiplicative interaction term (TyG × SHR) was incorporated into the regression models. Although the interaction term reached statistical significance in Models 1–3, the association was attenuated after full adjustment and was no longer statistically significant in Model 4 (OR = 0.67, 95% CI: 0.44–1.01, *p* = 0.059), indicating no clear evidence of multiplicative interaction between TyG and SHR in the fully adjusted model.

**Table 4:** Joint association of TyG and SHR with delirium risk in critically ill surgical patients.

Variables	Model 1		Model 2		Model 3		Model 4	
	OR (95%CI)	*p*	OR (95%CI)	*p*	OR (95%CI)	*p*	OR (95%CI)	*p*
T								
1	1.00 (Reference)		1.00 (Reference)		1.00 (Reference)		1.00 (Reference)	
2	2.12 (1.17 ~ 3.87)	0.014	2.09 (1.14 ~ 3.83)	0.017	1.94 (1.02 ~ 3.67)	0.043	2.06 (1.06 ~ 4.01)	0.033
3	4.02 (2.32 ~ 6.95)	<0.001	4.13 (2.38 ~ 7.18)	<0.001	2.85 (1.61 ~ 5.07)	<0.001	2.39 (1.31 ~ 4.37)	0.005
4	6.63 (4.01 ~ 10.94)	<0.001	6.45 (3.90 ~ 10.68)	<0.001	4.09 (2.36 ~ 7.09)	<0.001	3.57 (2.00 ~ 6.36)	<0.001
Interaction term (TyG × SHR)	0.69 (0.49 ~ 0.95)	0.024	0.67 (0.48 ~ 0.94)	0.018	0.67 (0.46 ~ 0.96)	0.032	0.67 (0.44 ~ 1.01)	0.059

OR, odds ratio; CI, confidence interval.

Model 1: Crude.

Model 2: Adjust: gender, age, sbp, weight, SpO₂.

Model 3: Adjust: gender, age, sbp, weight, spo2, hemoglobin, platelet, WBC, RBC, chloride, calcium, aniongap, bicarbonate, HDL, TC, LDL, SOFA, SAPSII.

Model 4: Adjust: cerebrovascular, dementia, gender, opioidanalgesics_used, insulin_used, benzodiazepines_used, vasoactive_used, CRRT, age, sbp, weight, SpO₂, hemoglobin, platelet, WBC, RBC, chloride, calcium, aniongap, bicarbonate, HDL, TC, LDL, SOFA, SAPSII.

**Figure 3:**
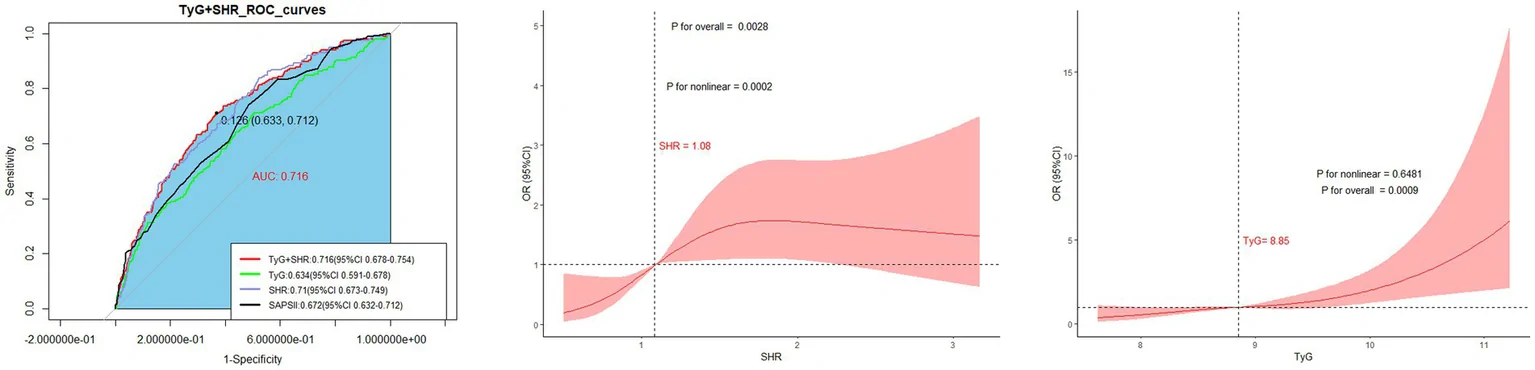
Dose–response relationships and discriminatory performance of TyG and SHR for delirium.

As shown in Figure 3, ROC curve analysis revealed that the combined TyG-SHR indicator had moderate discriminatory ability for delirium, with an AUC of 0.716, and outperformed either individual indicator alone, implying that combining these two indices may improve delirium risk identification. RCS analysis showed a significant nonlinear association between SHR and delirium risk (P for overall = 0.0028; P for nonlinear = 0.0002). In contrast, TyG showed a significant linear positive association with delirium risk (P for overall = 0.0009; P for nonlinear = 0.6481). These findings suggest that both TyG and SHR can reflect elevated delirium risk, although SHR may exhibit a more complex nonlinear dose–response relationship.

### 3.4 Subgroup analyses

**Figure 4:**
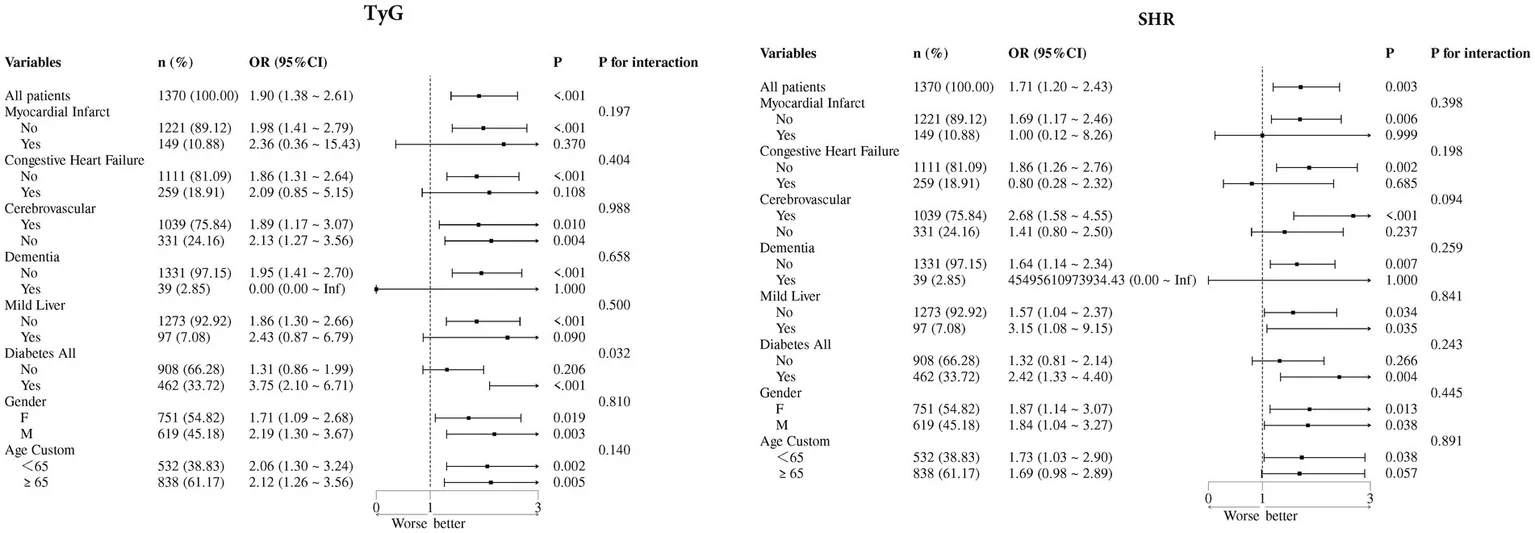
Subgroup analyses of the associations of TyG and SHR with delirium risk.

To further explore the associations of the TyG index and SHR with delirium among critically ill surgical patients across different clinical characteristics, we conducted multiple subgroup analyses. As shown in Figure 4, the TyG index showed a positive association in most subgroups, and a significant interaction was observed only in the subgroup stratified by diabetes status (*p* for interaction = 0.032). Specifically, this association was stronger among patients with diabetes (OR = 3.75, 95%CI: 2.10–6.71), whereas it did not reach statistical significance among patients without diabetes. In contrast, the direction of the SHR association was generally consistent across subgroups, and none of the interaction tests reached statistical significance (all *p* for interaction >0.05), indicating that the association between SHR and delirium was relatively stable. Although the estimated effect of SHR appeared stronger in patients with cerebrovascular disease, no significant interaction was observed.

### 3.5 Sensitivity analysis

In the first sensitivity analysis, we excluded patients with severe anemia and those whose HbA1c, triglyceride, glucose, or maximum glucose measurements were obtained more than 24 h after ICU admission. TyG and SHR were subsequently recalculated using these available early metabolic measurements. As shown in Supplementary Table S3, the associations between combined TyG-SHR groups and delirium remained largely unchanged. In the fully adjusted model (Model 4), patients in the highest combined TyG-SHR group (T4) continued to exhibit a significantly increased odds of delirium compared with the reference group (OR 4.60, 95% CI 1.99–10.61, *p* = 0.001), while the T3 group also remained significantly associated with delirium odds (OR 4.30, 95% CI 1.91–9.69, *p* < 0.001).

In the second sensitivity analysis, we excluded all patients with missing values in key study variables and repeated the analyses using a complete-case cohort without multiple imputation. As shown in Supplementary Table S4, the direction and magnitude of the associations were generally consistent with those observed in the primary analysis. In the fully adjusted model, the highest combined TyG-SHR group remained independently associated with an increased odds of delirium (OR 3.05, 95% CI 1.56–5.95, *p* = 0.001), and the T3 group also remained statistically significant (OR 2.14, 95% CI 1.08–4.24, *p* = 0.029).

Overall, the results of both sensitivity analyses were broadly consistent with those of the primary analyses, supporting the robustness of the observed association between combined TyG-SHR status and delirium odds in critically ill surgical and trauma patients.

### 3.6 Machine learning-based predictive models

Based on Boruta feature selection, a total of 23 features were included for model development, including stress hyperglycemia ratio (SHR), triglyceride-glucose index (TyG), Simplified Acute Physiology Score II (SAPS II), Sequential Organ Failure Assessment score (SOFA), continuous renal replacement therapy (CRRT), low-density lipoprotein cholesterol (LDL), total cholesterol (TC), high-density lipoprotein cholesterol (HDL), vasoactive drug use, benzodiazepine use, insulin use, opioid analgesic use, bicarbonate, anion gap, calcium, chloride, red blood cell count, white blood cell count, platelet count, hemoglobin, oxygen saturation, body weight, and systolic blood pressure. The dataset was randomly divided into a training set (70%) and a testing set (30%). Baseline characteristics of the training and testing cohorts are presented in Supplementary Table S5. In the training set, nine machine learning models were created using delirium as the outcome, which were then tested in the testing set. All reported ROC analyses, AUC values, calibration curves, and decision curve analyses (DCA) evaluations were derived from the test set.

**Figure 5:**
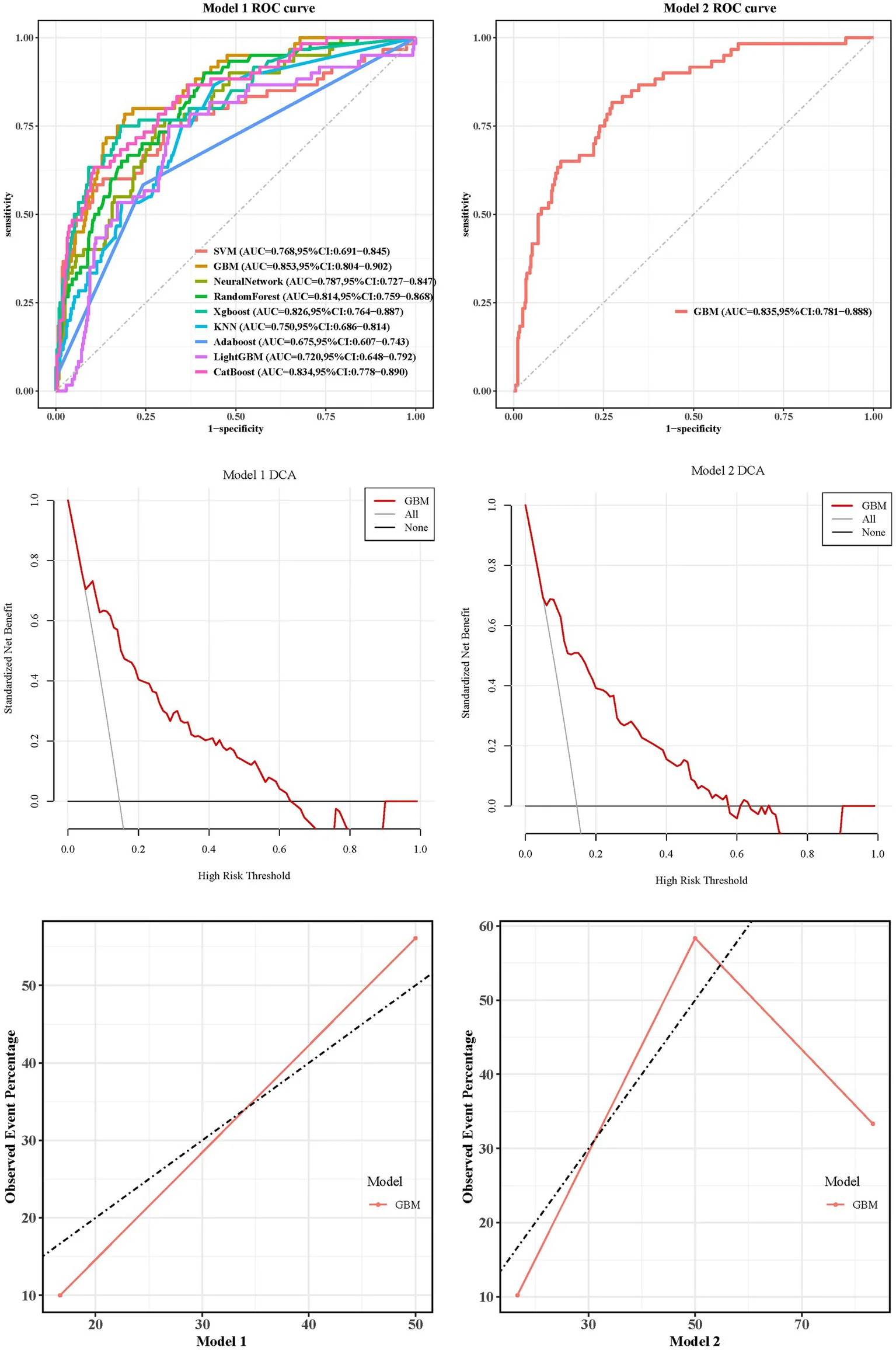
Comparative performance of nine machine learning models in predicting delirium among surgical critically ill patients.

As shown in Figure 5, the gradient boosting machine (GBM) model achieved the best performance, with an AUC of 0.853 (95%CI: 0.804–0.902), outperforming CatBoost (AUC = 0.834), XGBoost (AUC = 0.826), random forest (AUC = 0.814), neural network (AUC = 0.787), SVM (AUC = 0.768), KNN (AUC = 0.750), LightGBM (AUC = 0.720), and AdaBoost (AUC = 0.675). As a result, GBM was chosen as the best model for further examination.

To further evaluate the incremental predictive value of TyG and SHR, we defined the GBM model including all 23 Boruta-selected features as prediction model 1, and then constructed prediction model 2 using the remaining 21 variables after excluding TyG and SHR. The results showed that after removing TyG and SHR, the discriminatory ability of the model decreased, with the AUC declining from 0.853 to 0.835. We compared a base GBM model excluding these biomarkers with a full model including both biomarkers on the independent test set (Supplementary Table S6). Although the addition of TyG and SHR increased the AUC from 0.835 to 0.853, the improvement was not statistically significant (ΔAUC = 0.018, *p* = 0.217). Likewise, the IDI did not reach statistical significance (IDI = 0.008, 95% CI −0.009 to 0.025, *p* = 0.320). However, the continuous NRI was significantly positive (NRI = 0.363, 95% CI 0.092–0.613, *p* = 0.007), suggesting improved patient reclassification, primarily driven by more accurate identification of patients who did not subsequently develop delirium. Meanwhile, the calibration curve showed that the model including TyG and SHR fitted the ideal diagonal line more closely, whereas the model without TyG and SHR deviated further from the ideal line. Decision curve analysis (DCA) also indicated that the model including TyG and SHR provided greater clinical net benefit over a wider range of threshold probabilities. These results suggest that the inclusion of TyG and SHR can further improve the early identification of critically ill surgical patients at high risk of delirium.

**Figure 6:**
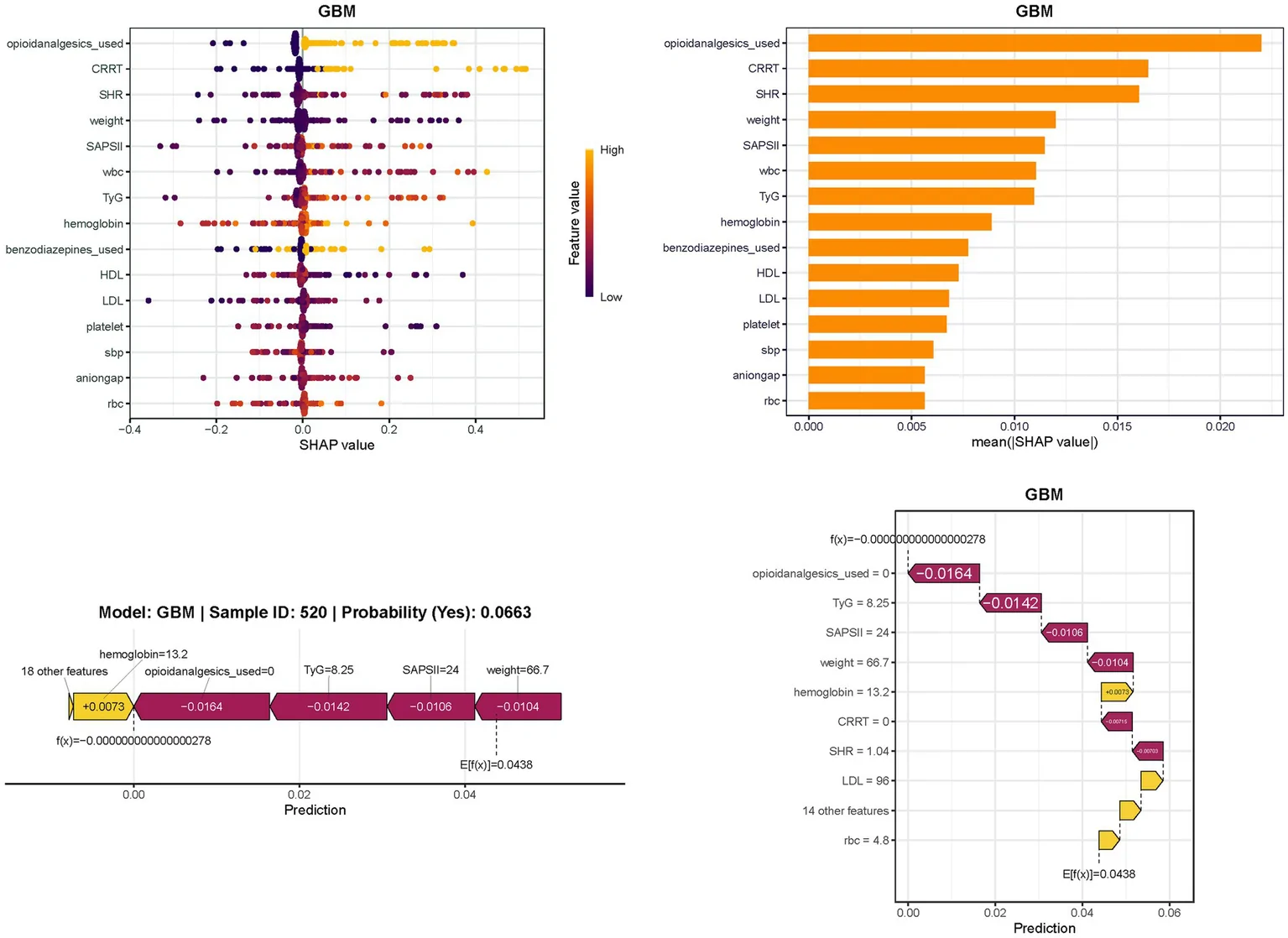
SHAP-based interpretation of the optimal GBM model for delirium prediction.

To enhance model interpretability, we further performed SHAP analysis for the optimal GBM model (Figure 6). SHAP analysis ranked opioid analgesic use, CRRT, SHR, body weight, SAPS II, TyG, hemoglobin, benzodiazepine use, HDL, and LDL as the ten most influential predictors in the final GBM model (Figure 6). The beeswarm plot further demonstrated that higher opioid analgesic use, CRRT, SHR, SAPS II, and TyG levels generally tended to drive the model prediction toward a higher risk of delirium.

In addition, we further presented a local SHAP interpretation for an individual sample. For sample 520, the predicted probability of delirium was 0.0663. Local interpretation showed that no opioid analgesic use, a TyG value of 8.25, a SAPS II score of 24, and a body weight of 66.7 kg were the main factors reducing the predicted risk for this sample. Both the force plot and waterfall plot showed that the largest negative contributions were, in descending order, no opioid analgesic use (−0.0164), a TyG value of 8.25 (−0.0142), a SAPS II score of 24 (−0.0106), and a body weight of 66.7 kg (−0.0104); in addition, no CRRT and an SHR value of 1.04 also showed negative contributions. In contrast, the positive contributions were relatively small, mainly arising from a hemoglobin level of 13.2 g/dL (+0.0073), as well as LDL cholesterol, red blood cell count, and a few other variables. These findings suggest that this sample was classified as low risk not because it completely lacked risk factors, but because the combined effects of multiple features that lowered the model output outweighed the smaller positive risk contributions.

## 4 Discussion

Based on the MIMIC-IV database, this study systematically evaluated the associations of the triglyceride-glucose index (TyG) and stress hyperglycemia ratio (SHR) with delirium in critically ill surgical patients. The main findings were as follows. First, both TyG and SHR were independently associated with delirium odds after full adjustment for confounding factors. Second, when TyG and SHR were jointly elevated, the odds of delirium increased in a stepwise manner, suggesting a possible cumulative effect. Third, dose–response analyses showed that TyG was mainly linearly positively associated with delirium odds, whereas SHR exhibited a significant nonlinear association with delirium odds. Fourth, in the machine learning analyses, incorporating TyG and SHR improved patient odds reclassification and model calibration. Overall, our findings suggest that TyG and SHR reflect delirium odds from two complementary dimensions, namely baseline metabolic vulnerability and acute stress-related metabolic dysregulation.

Our findings are generally consistent with previous studies on metabolic abnormalities and adverse outcomes in critically ill patients, while further extending this line of research to delirium in critically ill surgical patients, a specific clinical endpoint for which evidence remains relatively limited. Previous studies have focused more on the associations of single blood glucose measures, stress hyperglycemia, or TyG with outcomes such as mortality, cardiovascular events, and infection (20, 26, 27), whereas studies specifically addressing delirium in surgical/trauma ICU populations are relatively scarce. Compared with these studies, the present study has several distinctive features. We simultaneously examined the independent effects of TyG and SHR, further evaluated their joint associations, nonlinear relationships, and consistency across clinically relevant subgroups, and integrated machine learning models with SHAP-based interpretation to enhance predictive assessment and model interpretability. Therefore, our findings provide more comprehensive evidence linking chronic metabolic vulnerability and acute stress-related hyperglycemia to delirium risk in critically ill surgical and trauma patients, extending previous studies that primarily focused on a single metabolic marker.

From a pathophysiological perspective, the associations of TyG and SHR with delirium are biologically plausible. Delirium is considered to result from the combined effects of multiple factors, among which neuroinflammation, blood–brain barrier disruption, cerebral microcirculatory dysfunction, oxidative stress, and neurotransmitter imbalance are widely recognized as core mechanisms (4, 28, 29). As a surrogate marker of insulin resistance and baseline metabolic abnormality, TyG reflects disturbances in lipid-glucose metabolism, chronic low-grade inflammation, oxidative stress, and endothelial dysfunction. These abnormalities may increase central nervous system vulnerability to perioperative and critical illness-related stress by impairing cerebral microvascular endothelial function, disrupting blood–brain barrier integrity, and interfering with neuronal energy metabolism (30–32). In other words, elevated TyG, as an indicator of chronic metabolic abnormality, may provide the “baseline susceptibility” for the development of delirium.

In contrast, SHR emphasizes the degree of abnormal hyperglycemia relative to prior glycemic background during acute illness and therefore better reflects acute stress burden rather than merely poor chronic glycemic control. Stress hyperglycemia is usually accompanied by activation of the sympathetic-neuroendocrine axis, increased release of pro-inflammatory cytokines, and aggravated insulin resistance, thereby resulting in more pronounced metabolic imbalance (33, 34). Previous studies have shown that hyperglycemia may exacerbate central nervous system inflammatory responses and neuronal injury by enhancing oxidative stress, promoting microglial activation and glycolytic reprogramming, damaging cerebral microvascular endothelial cell function, and disrupting blood–brain barrier integrity, thereby amplifying inflammatory cascades and oxidative stress (35–38). On this basis, elevated SHR may represent not only a metabolic manifestation of acute critical illness stress but also a pathological state more likely to trigger amplified neuroinflammation and neural dysfunction. Recent studies have also observed that elevated SHR is associated with an increased risk of delirium in patients undergoing CABG and in elderly surgical ICU populations (17, 39), which further supports the clinical interpretation of SHR as a marker of “acute disequilibrium.”

Therefore, although both TyG and SHR are associated with delirium, they represent different but complementary pathological dimensions. The former primarily reflects chronic vulnerability related to long-term insulin resistance and reduced baseline metabolic reserve, whereas the latter more directly reflects the intensity of acute metabolic disequilibrium corresponding to relative hyperglycemia during critical illness. Importantly, our findings suggest that the concurrent elevation of TyG and SHR identifies a subgroup of patients at particularly high risk of delirium. However, because the multiplicative interaction term did not reach statistical significance in the fully adjusted model, these findings should not be interpreted as evidence of a biological interaction or synergistic effect between the two biomarkers. Elevated TyG may indicate a pre-existing state of insulin resistance, endothelial dysfunction, impaired cerebrovascular autoregulation, and increased blood–brain barrier susceptibility. Against this vulnerable background, acute stress hyperglycemia reflected by elevated SHR may further exacerbate oxidative stress, neuroinflammatory activation, mitochondrial dysfunction, and microvascular injury. The convergence of these chronic and acute pathological processes may amplify blood–brain barrier disruption, facilitate the penetration of peripheral inflammatory mediators into the central nervous system, promote microglial activation, and disrupt neurotransmitter homeostasis. Instead, the concurrent elevation of TyG and SHR may represent the coexistence of chronic metabolic vulnerability and acute stress-related metabolic dysregulation. Patients exhibiting elevations in both biomarkers had the highest observed risk of delirium, suggesting that their combined assessment may improve metabolic risk stratification rather than demonstrating a biological interaction.

Several limitations should also be recognized. First, this study was based on a single-center critical care database and used a retrospective observational design, thus causation cannot be established and residual confounding may still present. Second, in critically ill surgical and trauma ICU patients, early metabolic measurements may be substantially influenced by acute pathophysiological stress and by treatments initiated before or shortly after ICU admission, including fluid resuscitation, glucose-containing solutions, vasoactive agents, insulin therapy, and enteral or parenteral nutritional support in the emergency department or operating room. Therefore, TyG calculated from early in-hospital measurements may not fully represent pre-existing fasting metabolic status or chronic insulin resistance. Third, SHR was constructed using the first in-hospital HbA1c value and the maximum glucose value within 24 h after ICU admission. Although biologically reasonable, this approach cannot fully replace dynamic glucose monitoring. TyG and SHR should be interpreted as metabolic risk markers obtained during early critical illness. Moreover, because SHR incorporated the maximum glucose value within the first 24 h after ICU admission, it may have been influenced by treatment intensity, frequency of glucose monitoring, and evolving critical illness, thereby introducing potential treatment-related or surveillance-related bias. In addition, because HbA1c testing was performed according to routine clinical practice rather than a standardized study protocol, restricting the study population to patients with available HbA1c measurements may have introduced selection bias. Fourth, although delirium was assessed in a structured manner using CAM-ICU, hypoactive delirium or transient episodes occurring between assessments may still have been underestimated. Fifth, although several key medications and organ support variables were included, not all potential interventions could be captured, such as analgesic/sedative doses, nutritional support strategies, and specific perioperative management measures. Sixth, although subgroup analyses were performed to explore potential effect modification, these analyses were exploratory in nature and were not the primary focus of this study. No additional multivariable adjustment was performed within individual subgroups, and some strata contained relatively small numbers of patients. Therefore, the observed heterogeneity in certain subgroups, including the myocardial infarction subgroup, should be interpreted cautiously and may reflect random variation, limited statistical power, or residual confounding rather than true biological differences. Finally, the machine learning models underwent only internal train-test validation, without independent external validation cohorts; thus, their generalizability still requires further evaluation.

Given that TyG and SHR can be calculated from routinely available laboratory measurements obtained during ICU admission, these biomarkers may be incorporated into early delirium screening and risk stratification workflows. Patients presenting with concurrent elevations in both TyG and SHR may represent a particularly high-risk population who could benefit from closer neurological monitoring and targeted delirium prevention strategies. Future studies are warranted to determine whether a simplified bedside risk score integrating these biomarkers could further improve clinical applicability.

## 5 Conclusion

This study showed that, in critically ill surgical patients, both the TyG index and SHR were independently associated with delirium, and that concurrent elevation of both indices was associated with a further increase in delirium odds. TyG showed a predominantly linear positive association with delirium odds, whereas SHR exhibited a significant nonlinear dose–response relationship. Overall, as readily obtainable metabolic indicators, TyG and SHR may serve as useful complementary tools for the early identification and odds stratification of delirium in critically ill surgical patients; however, their clinical utility still requires further confirmation through prospective studies and external validation.

## Data Availability

Publicly available datasets were analyzed in this study. The datasets analyzed during the current study are available in the MIMIC-IV database (version 3.1) on PhysioNet and can be accessed upon completion of the required credentialing process.
