# Supplementary material for: Nutritional-metabolic risk stratification for delirium in surgical critically ill patients: joint associations of the triglyceride–glucose index and stress hyperglycemia ratio
Source: Front Nutr. 2026 Sep 9;13:1875967. doi: 10.3389/fnut.2026.1875967 (PMC13599493; doi:10.3389/fnut.2026.1875967)
Supplement: Supplementary file 1 [file Table_1.DOCX]

**Supplementary TableS1: Hyperparameter settings of the nine machine learning models**

| **Model** | **Algorithm** | **Hyperparameter setting** |
| --- | --- | --- |
| SVM | Support vector machine | sigma = 0.001; C = 10 |
| GBM | Gradient boosting machine | n.trees = 50; interaction.depth = 5; shrinkage = 0.05; n.minobsinnode = 10 |
| NNET | Neural network | size = 6; decay = 0.6 |
| RF | Random forest | mtry = 11; numRandomCuts = 3 |
| XGBoost | Extreme gradient boosting | nrounds = 10; max_depth = 3; eta = 0.001; gamma = 0.5; colsample_bytree = 0.5; min_child_weight = 1; subsample = 0.6 |
| KNN | k-nearest neighbors | kmax = 12; distance = 1; kernel = optimal |
| AdaBoost | Adaptive boosting | mfinal = 2; maxdepth = 2; coeflearn = Zhu |
| LightGBM | Light gradient boosting machine | objective = binary; metric = auc; min_data = 1; learning_rate = 1.0; num_threads = 2; force_col_wise = TRUE; nrounds = 5; early_stopping_rounds = 3 |
| CatBoost | Categorical boosting | iterations = 100; use_best_model = TRUE; eval_metric = AUC; ignored_features = c(4, 9); border_count = 32; depth = 5; learning_rate = 0.03; random_seed = 123 |
