# Supplementary material for: Nutritional-metabolic risk stratification for delirium in surgical critically ill patients: joint associations of the triglyceride–glucose index and stress hyperglycemia ratio
Source: Front Nutr. 2026 Sep 9;13:1875967. doi: 10.3389/fnut.2026.1875967 (PMC13599493; doi:10.3389/fnut.2026.1875967)
Supplement: Supplementary file 2 [file Table_2.DOCX]

Supplementary Table S2. Proportions of missing values before multiple imputation and variance inflation factor (VIF) results for key study variables.

| Variable | VIF | Missing proportion (%) |
| --- | --- | --- |
| SHR | 1.50562 | 0 |
| TyG | 1.839478 | 0 |
| gender | 1.290863 | 0 |
| age | 1.939214 | 0 |
| SAPSII | 2.186677 | 0 |
| SOFA | 1.716941 | 0.07 |
| CRRT | 1.360821 | 0 |
| LDL | 3.578584 | 10.88 |
| TC | 4.31556 | 7.45 |
| HDL | 1.911883 | 10.07 |
| Vasoactive used | 1.312497 | 0 |
| Benzodiazepines used | 1.099589 | 0 |
| Insulin used | 1.071938 | 0 |
| Opioid analgesics used | 1.347335 | 0 |
| bicarbonate | 2.042204 | 3.87 |
| Anion gap | 1.989491 | 3.94 |
| calcium | 1.395672 | 12.12 |
| chloride | 1.478834 | 2.99 |
| RBC | 4.923199 | 1.24 |
| WBC | 1.357337 | 1.17 |
| platelet | 1.293608 | 0.51 |
| hemoglobin | 5.03637 | 1.17 |
| spo2 | 1.056238 | 0.07 |
| weight | 1.158408 | 0.95 |
| SBP | 1.168247 | 0.07 |
| dementia | 1.048408 | 0 |
| cerebrovascular | 1.385818 | 0 |
