# Supplementary material for: Nutritional-metabolic risk stratification for delirium in surgical critically ill patients: joint associations of the triglyceride–glucose index and stress hyperglycemia ratio
Source: Front Nutr. 2026 Sep 9;13:1875967. doi: 10.3389/fnut.2026.1875967 (PMC13599493; doi:10.3389/fnut.2026.1875967)
Supplement: Supplementary file 3 [file Table_3.DOCX]

### Supplementary Table S3. Sensitivity analysis of the association between combined TyG-SHR groups and delirium after excluding patients with severe anemia and metabolic measurements obtained more than 24 hours after ICU admission.

| Variables | Model1 | |  | Model2 | |  | Model3 | |  | Model4 | |
| --- | --- | --- | --- | --- | --- | --- | --- | --- | --- | --- | --- |
|  | OR (95%CI) | *P* |  | OR (95%CI) | *P* |  | OR (95%CI) | *P* |  | OR (95%CI) | *P* |
| T |  |  |  |  |  |  |  |  |  |  |  |
| 1 | 1.00 (Reference) |  |  | 1.00 (Reference) |  |  | 1.00 (Reference) |  |  | 1.00 (Reference) |  |
| 2 | 1.83 (0.76 ~ 4.44) | 0.179 |  | 1.82 (0.75 ~ 4.43) | 0.188 |  | 2.03 (0.81 ~ 5.09) | 0.133 |  | 2.17 (0.84 ~ 5.60) | 0.111 |
| 3 | 5.86 (2.74 ~ 12.55) | <.001 |  | 6.23 (2.90 ~ 13.40) | <.001 |  | 4.56 (2.06 ~ 10.08) | <.001 |  | 4.30 (1.91 ~ 9.69) | <.001 |
| 4 | 6.31 (3.02 ~ 13.15) | <.001 |  | 6.24 (2.97 ~ 13.12) | <.001 |  | 4.52 (2.02 ~ 10.11) | <.001 |  | 4.60 (1.99 ~ 10.61) | <.001 |

OR: Odds Ratio, CI: Confidence Interval

Model1: Crude

Model2: Adjust: gender, age, sbp, weight, spo2

Model3: Adjust: gender, age, sbp, weight, spo2, hemoglobin, platelet, wbc, rbc, chloride, calcium, aniongap, bicarbonate, HDL, TC, LDL, SOFA, SAPSII

Model4: Adjust: cerebrovascular, dementia, gender, opioidanalgesics_used, insulin_used, benzodiazepines_used, vasoactive_used, CRRT, age, sbp, weight, spo2, hemoglobin, platelet, wbc, rbc, chloride, calcium, aniongap, bicarbonate, HDL, TC, LDL, SOFA, SAPSII
