# Supplementary material for: Nutritional-metabolic risk stratification for delirium in surgical critically ill patients: joint associations of the triglyceride–glucose index and stress hyperglycemia ratio
Source: Front Nutr. 2026 Sep 9;13:1875967. doi: 10.3389/fnut.2026.1875967 (PMC13599493; doi:10.3389/fnut.2026.1875967)
Supplement: Supplementary file 4 [file Table_4.DOCX]

### Supplementary Table S4. Complete-case sensitivity analysis for the association between combined TyG-SHR groups and delirium risk after excluding patients with missing values in key study variables.

| Variables | Model1 | |  | Model2 | |  | Model3 | |  | Model4 | |
| --- | --- | --- | --- | --- | --- | --- | --- | --- | --- | --- | --- |
|  | OR (95%CI) | *P* |  | OR (95%CI) | *P* |  | OR (95%CI) | *P* |  | OR (95%CI) | *P* |
| T |  |  |  |  |  |  |  |  |  |  |  |
| 1 | 1.00 (Reference) |  |  | 1.00 (Reference) |  |  | 1.00 (Reference) |  |  | 1.00 (Reference) |  |
| 2 | 1.60 (0.78 ~ 3.29) | 0.196 |  | 1.64 (0.80 ~ 3.38) | 0.179 |  | 1.74 (0.81 ~ 3.73) | 0.157 |  | 1.64 (0.75 ~ 3.58) | 0.216 |
| 3 | 3.63 (1.93 ~ 6.83) | <.001 |  | 3.64 (1.93 ~ 6.87) | <.001 |  | 2.42 (1.24 ~ 4.71) | 0.009 |  | 2.14 (1.08 ~ 4.24) | 0.029 |
| 4 | 5.07 (2.81 ~ 9.13) | <.001 |  | 5.12 (2.83 ~ 9.24) | <.001 |  | 3.19 (1.67 ~ 6.10) | <.001 |  | 3.05 (1.56 ~ 5.95) | 0.001 |

OR: Odds Ratio, CI: Confidence Interval

Model1: Crude

Model2: Adjust: gender, age, sbp, weight, spo2

Model3: Adjust: gender, age, sbp, weight, spo2, hemoglobin, platelet, wbc, rbc, chloride, calcium, aniongap, bicarbonate, HDL, TC, LDL, SOFA, SAPSII

Model4: Adjust: cerebrovascular, dementia, gender, opioidanalgesics_used, insulin_used, benzodiazepines_used, vasoactive_used, CRRT, age, sbp, weight, spo2, hemoglobin, platelet, wbc, rbc, chloride, calcium, aniongap, bicarbonate, HDL, TC, LDL, SOFA, SAPSII
