# Supplementary material for: Nutritional-metabolic risk stratification for delirium in surgical critically ill patients: joint associations of the triglyceride–glucose index and stress hyperglycemia ratio
Source: Front Nutr. 2026 Sep 9;13:1875967. doi: 10.3389/fnut.2026.1875967 (PMC13599493; doi:10.3389/fnut.2026.1875967)
Supplement: Supplementary file 5 [file Table_5.DOCX]

Supplementary Table S5. Baseline characteristics of the training and testing cohorts used for machine learning model development and validation.

| Variables | level | Testing cohort | Training cohort | | p |
| --- | --- | --- | --- | --- | --- |
| n |  | 411 | 959 |  | |
| SHR |  | 1.19 (0.46) | 1.20 (0.50) | 0.914 | |
| TyG |  | 8.93 (0.71) | 8.96 (0.73) | 0.446 | |
| SAPSII |  | 32.58 (12.62) | 31.90 (11.90) | 0.34 | |
| SOFA |  | 1.22 (2.04) | 1.13 (1.75) | 0.39 | |
| CRRT | No | 393 (95.6) | 930 (97.0) | 0.271 | |
|  | Yes | 18 (4.4) | 29 (3.0) |  | |
| LDL |  | 86.64 (40.32) | 88.53 (41.20) | 0.434 | |
| TC |  | 159.81 (55.24) | 161.87 (59.54) | 0.549 | |
| HDL |  | 46.13 (17.65) | 45.42 (17.47) | 0.487 | |
| vasoactive used | No | 346 (84.2) | 792 (82.6) | 0.519 | |
|  | Yes | 65 (15.8) | 167 (17.4) |  | |
| benzodiazepines used | No | 311 (75.7) | 699 (72.9) | 0.315 | |
|  | Yes | 100 (24.3) | 260 (27.1) |  | |
| insulin used | No | 108 (26.3) | 265 (27.6) | 0.652 | |
|  | Yes | 303 (73.7) | 694 (72.4) |  | |
| Opioid analgesics used | No | 268 (65.2) | 640 (66.7) | 0.627 | |
|  | Yes | 143 (34.8) | 319 (33.3) |  | |
| bicarbonate |  | 23.39 (3.80) | 23.75 (3.94) | 0.115 | |
| Anion gap |  | 14.64 (3.32) | 14.59 (3.64) | 0.797 | |
| calcium |  | 8.61 (0.85) | 8.70 (0.73) | 0.052 | |
| chloride |  | 103.94 (5.43) | 103.44 (5.45) | 0.114 | |
| RBC |  | 4.07 (0.74) | 4.05 (0.73) | 0.728 | |
| WBC |  | 10.93 (5.40) | 10.89 (5.11) | 0.882 | |
| platelet |  | 222.26 (93.67) | 228.15 (96.00) | 0.294 | |
| hemoglobin |  | 12.28 (2.20) | 12.10 (2.17) | 0.164 | |
| spo2 |  | 97.22 (2.77) | 97.28 (2.85) | 0.717 | |
| weight |  | 83.26 (25.01) | 82.57 (41.33) | 0.756 | |
| SBP |  | 139.09 (25.31) | 141.58 (25.53) | 0.097 | |
