# Supplementary material for: Nutritional-metabolic risk stratification for delirium in surgical critically ill patients: joint associations of the triglyceride–glucose index and stress hyperglycemia ratio
Source: Front Nutr. 2026 Sep 9;13:1875967. doi: 10.3389/fnut.2026.1875967 (PMC13599493; doi:10.3389/fnut.2026.1875967)
Supplement: Supplementary file 6 [file Table_6.DOCX]

Supplementary Table S6. Incremental predictive and reclassification performance of adding TyG and SHR to the GBM model on the independent test set.

| Item | Estimate | Lower_95_CI | Upper_95_CI | P |
| --- | --- | --- | --- | --- |
| Base model without TyG and SHR: AUC | 0.8347 | 0.7809 | 0.8884 |  |
| Full model with TyG and SHR: AUC | 0.8528 | 0.8036 | 0.9019 |  |
| Delta AUC | 0.0181 | -0.0091 | 0.048 | 0.217 |
| Continuous NRI | 0.3632 | 0.0924 | 0.6132 | 0.00717 |
| NRI for events | 0.1667 | -0.082 | 0.4099 | 0.188 |
| NRI for non-events | 0.1966 | 0.0914 | 0.2918 | 0.00017 |
| IDI | 0.0083 | -0.0085 | 0.0247 | 0.32 |
